# Retraction: Chitinase 1 regulates pulmonary fibrosis by modulating TGF-β/SMAD7 pathway via TGFBRAP1 and FOXO3

**DOI:** 10.26508/lsa.202402987

**Published:** 2024-08-29

**Authors:** Chang-Min Lee, Chuan-Hua He, Jin Wook Park, Jae Hyun Lee, Suchitra Kamle, Bing Ma, Bedia Akosman, Roberto Cortez, Emily Chen, Yang Zhou, Erica L Herzog, Changwan Ryu, Xueyan Peng, Ivan O Rosas, Sergio Poli, Carol Feghali Bostwick, Augustine M Choi, Jack A Elias, Chun Geun Lee

**Affiliations:** 1 Molecular Microbiology and Immunology, Brown University , Providence, RI, USA; 2 Department of Internal Medicine, Yonsei University College of Medicine, Seoul, South Korea; 3 Section of Pulmonary, Critical Care, and Sleep Medicine, Department of Internal Medicine, Yale School of Medicine, New Haven, CT, USA; 4 Brigham and Women’s Hospital, Boston, MA, USA; 5 Department of Medicine, College of Medicine, Medical University of South Carolina , Charleston, SC, USA; 6 Weill Cornell Medicine Pulmonary and Critical Care Medicine, New York, NY, USA; 7 Division of Medicine and Biological Sciences, Brown University , Warren Alpert School of Medicine, Providence, RI, USA

## Abstract

Chitinase 1 (CHIT1) plays a role in the pathogenesis of pulmonary fibrosis by modulating canonical and noncanonical TGF-β signaling via interaction with TGFBRAP1 and FOXO3. These findings highlight the CHIT1/SMAD7 axis as a potential biomarker and therapeutic target of pulmonary fibrosis.

Article: Lee C-M, He C-H, Park JW, Lee JH, Kamle S, Ma B, Akosman B, Cotez R, Chen E, Zhou Y, Herzog EL, Ryu C, Peng X, Rosas IO, Poli S, Bostwick CF, Choi AM, Elias JA, Lee CG (2019 May 13) Chitinase 1 regulates pulmonary fibrosis by modulating TGF-β/SMAD7 pathway via TGFBRAP1 and FOXO3. Life Sci Alliance 2(3): e201900350. doi: 10.26508/lsa.201900350. PMID: 31085559.

The authors wish to retract this article because concerns have been raised regarding the contents and labeling of some of the figures in this article, including Figs 2D, 3A, and 4A. These figures appear to include duplicate data or to incorporate separate experiments that are not accurately labeled. Some of the immunoblot data cannot be verified from the records available. Although we believe that the conclusions of the article are still valid, in light of the inaccuracies in these figures, we consider that the appropriate course of action is to retract the article. All the authors agree with the retraction.

